# Combinations of Independent Motor Functional Independence Measure Items Associated With Home Discharge in Stroke Patients in Post-acute Rehabilitation Wards: An UpSet Plot Analysis

**DOI:** 10.7759/cureus.73982

**Published:** 2024-11-19

**Authors:** Dai Nakaizumi, Keita Uchiyama, Kei Washida, Shingo Miyata

**Affiliations:** 1 Department of Physical Therapy, Graduate Course of Rehabilitation Science, Division of Health Sciences, College of Medical, Pharmaceutical, and Health Sciences, Kanazawa University, Kanazawa, JPN; 2 Department of Rehabilitation, Japanese Red Cross Kanazawa Hospital, Kanazawa, JPN

**Keywords:** adl (activities of daily living), discharge to home, functional independence measure (fim), kaifukuki (convalescent) rehabilitation ward (krw), post-stroke rehabilitation, upset plot, visualization analysis

## Abstract

Objectives

Home discharge is a crucial goal for stroke patients in convalescent rehabilitation wards. While previous studies have identified relationships between Functional Independence Measure (FIM) scores and home discharge outcomes using conventional statistical methods, these analyses have limitations in visualizing combinations of independent FIM items. This study aimed to identify combinations of independent motor FIM items associated with home discharge in stroke patients using UpSet plots.

Methods

We analyzed 205 stroke patients admitted to a convalescent rehabilitation ward between April 2018 and March 2021. Independence in motor FIM items was defined as a score of 6 or higher. We used UpSet plots to visualize combinations of independent motor FIM items at discharge and identified major combinations (occurring in >7% of cases) in the home discharge group.

Results

Among 136 patients discharged to home, four major combinations of independent motor FIM items were identified: independence in all 13 items (22 patients, or 16.2%), independence in 12 items excluding stairs (18 patients, or 13.2%), independence in 10 items excluding bathing, tub transfer, and stairs (14 patients, or 10.3%), and dependence in all items (11 patients, or 8.1%). These combinations accounted for 47.8% of home discharge cases.

Conclusions

The visual analysis using UpSet plots revealed that, while higher levels of activities of daily living (ADL) independence increased the likelihood of home discharge, complete independence was not required. Dependence on specific items, such as bathing, tub transfer, and stairs, could be compensated through care services and environmental modifications. These findings provide practical insights for developing rehabilitation strategies and discharge planning.

## Introduction

In Japan, post-acute rehabilitation is provided in specialized rehabilitation units known as 'convalescent rehabilitation wards' (kaifukuki rehabilitation wards). This system was established in 2000 to bridge the gap between acute care and community-based rehabilitation [1]. Home discharge is a crucial goal for stroke patients in these convalescent rehabilitation wards. According to the 2024 revision of medical service fees in Japan, convalescent rehabilitation wards (grades 1-4) must achieve a minimum home discharge rate of 70% as a facility criterion [2]. This revision emphasizes the importance of providing high-quality rehabilitation services to facilitate home discharge for more patients.

The Functional Independence Measure (FIM) is widely used to assess activities of daily living (ADL) in stroke patients. The FIM consists of 13 motor items and 7 cognitive items, each scored on a seven-point scale (1-7) based on the level of independence. Literature reviews have identified various FIM-related factors associated with home discharge [3]. These factors include higher motor FIM scores [4-6], higher cognitive FIM scores [7], higher scores in both motor and cognitive FIM items [8,9], and higher total FIM scores [10,11]. Specific FIM items, such as upper body dressing and bowel management, have been identified as predictors of home discharge [12]. Additional studies have reported that toileting [13], self-care activities (eating, grooming, bathing, dressing), continence management (bladder, bowel), transfers (bed/chair/wheelchair, toilet, shower/tub), and locomotion (walking/wheelchair, stairs) [14] are also predictive factors for home discharge.

Previous studies have primarily used logistic regression analysis to examine the relationship between FIM scores and home discharge. This statistical method effectively evaluates how specific factors influence outcomes by revealing associations between total FIM scores or specific FIM items and home discharge probability. Recently, decision tree analysis has emerged as another analytical approach for studying FIM-related factors and predicting home discharge outcomes [12,15,16]. Decision tree analysis offers multiple advantages. First, it presents results in an easily interpretable tree structure. Second, it provides clear threshold values by systematically dividing data into subsets based on predictor variables. Third, it hierarchically organizes variables according to their relative importance, thereby clarifying relationships among multiple factors.

While logistic regression and decision tree analyses have revealed associations between FIM scores and home discharge, these findings have limitations in clinical application. In clinical practice, healthcare workers aim to improve total FIM scores but lack specific guidance on which items require improvement and to what extent. Furthermore, rehabilitation typically results in simultaneous improvements across multiple items, and enhancement of a single item may not necessarily lead to successful home discharge. Recent healthcare reforms have emphasized shorter hospital stays, requiring more efficient use of limited rehabilitation resources to effectively improve ADL capabilities and facilitate home discharge. Therefore, it is crucial to understand which combinations of independent FIM items are associated with successful home discharge and, conversely, which items need not be fully independent for home discharge to be feasible.

Traditional analytical methods have limitations in visualizing and extracting co-occurrence relationships among multiple FIM items. This study focuses on the UpSet plot technique, which can effectively visualize relationships among multiple factors. UpSet plots were developed to overcome the limitations of traditional visualization methods, such as Venn diagrams, which become visually complex and difficult to interpret when dealing with multiple sets [17,18]. While traditional Venn diagrams cannot effectively visualize relationships among 13 motor FIM items, UpSet plots enable detailed visualization of independence combinations in motor FIM items associated with home discharge. Therefore, this study aims to identify combinations of independent motor FIM items associated with home discharge in stroke patients using UpSet plots.

## Materials and methods

Study population

We analyzed 242 stroke patients admitted to our convalescent rehabilitation ward between April 2018 and March 2021. We excluded patients who died during hospitalization, were transferred to other hospitals due to acute deterioration, or were moved to acute care wards.

Data collection

We collected data from medical records, including patient demographics (age and sex), motor items of the FIM at discharge, and total FIM scores at discharge. The discharge FIM assessment was conducted by the patient's rehabilitation team, consisting of physical therapists, occupational therapists, speech therapists, and ward nurses. As the assessments were part of routine clinical practice, the evaluators were not randomized. There were no missing data in our dataset.

Outcome measures

We used the Japanese version of FIM(TM) version 3.0 [19,20], which has culturally relevant modifications for some of the items [21,22]. The FIM is an assessment tool that evaluates independence in ADL and consists of 18 items: 13 motor items and 5 cognitive items. The motor items comprise self-care (eating, grooming, bathing, dressing upper body, dressing lower body), sphincter control (toileting, bladder management, bowel management), transfers (bed/chair/wheelchair, toilet, tub/shower), and locomotion (walking/wheelchair, stairs). Each item is scored on a seven-point scale, ranging from 1 (total assistance) to 7 (complete independence). Previous studies have confirmed the reliability and validity of FIM for stroke patients [23]. In this study, we defined independence as a score of 6 or higher and dependence as a score of 5 or lower for each motor item.

Statistical analysis and visualization

We performed statistical analyses using R version 4.2.3 (R Foundation for Statistical Computing, Vienna, Austria). Patients were classified into home discharge and non-home discharge groups based on their discharge destination. We described patient characteristics using medians and interquartile ranges (IQRs) for continuous variables, and percentages (%) for categorical variables.

To visualize combinations of independent motor FIM items in the home discharge group, we created UpSet plots using the R package 'Complex Upset' [17,24]. We also created similar visualizations for the non-home discharge group for comparison. The UpSet plot comprises three main components: a dot plot showing combinations of FIM subitems at the bottom; a bar graph showing the number of independent patients for each FIM subitem on the left; and a bar graph showing the frequency of each combination at the top.

In the home discharge group (n = 136), we defined major combinations as those occurring in 10 or more cases (>7% of the total). This threshold was established based on preliminary analysis and clinical significance. The preliminary analysis revealed that the top four combinations accounted for 47.8% of the cumulative frequency. Additionally, we set 10 cases as the minimum clinically meaningful sample size, considering that combinations with lower frequencies might be incidental.

## Results

The study included 242 participants, and 37 were excluded because one died during hospitalization, 26 were transferred to other hospitals due to acute exacerbation, and 10 were transferred to acute care units. The final analysis included 205 patients, with 136 discharged home and 69 discharged to other facilities.

The median age of all participants was 78 years (IQR: 69-84 years). The home discharge group had a median age of 75 years (IQR: 67-83 years), while the non-home discharge group had a median age of 82 years (IQR: 73-87 years). Males comprised 54% of each group. The home discharge group showed higher scores in all motor FIM items and total FIM scores, compared to the non-home discharge group (Table 1).

**Table 1:** Characteristics of study population Data are presented as median (IQR) IQR, Interquartile range; FIM, Functional independent measure

Characteristic	Overall, N = 205	Home, N = 136	Non-home, N = 69
Age	78 (69-84)	75 (67-83)	82 (73-87)
Sex			
Male	110 (54%)	73 (54%)	37 (54%)
Female	95 (46%)	63 (46%)	32 (46%)
Eating	6 (5-7)	7 (5-7)	5 (4-5)
Grooming	6 (5-7)	7 (5-7)	4 (2-5)
Bathing	4 (3-6)	5 (4-7)	1 (1-4)
Dressing - upper body	5 (2-7)	7 (5-7)	2 (1-5)
Dressing - lower body	5 (1-7)	7 (5-7)	1 (1-4)
Toileting	6 (4-6)	6 (6-7)	2 (1-5)
Bladder management	7 (2-7)	7 (6-7)	1 (1-6)
Bowel management	6 (5-7)	6 (6-7)	4 (1-6)
Transfer (bed/chair/wheelchair)	6 (4-7)	6 (6-7)	4 (2-5)
Transfer (toilet)	6 (5-6)	6 (6-7)	4 (1-5)
Transfer (tub/shower)	5 (3-6)	5 (5-6)	1 (1-4)
Walk/wheelchair	5 (2-6)	6 (5-6)	1 (1-5)
Stairs	4 (1-5)	5 (3-5)	1 (1-2)
Motor FIM scores	71 (45-82)	78 (66-84)	35 (19-56)
Total FIM scores	98 (65-113)	108 (92-117)	59 (34-79)

**Figure 1:**
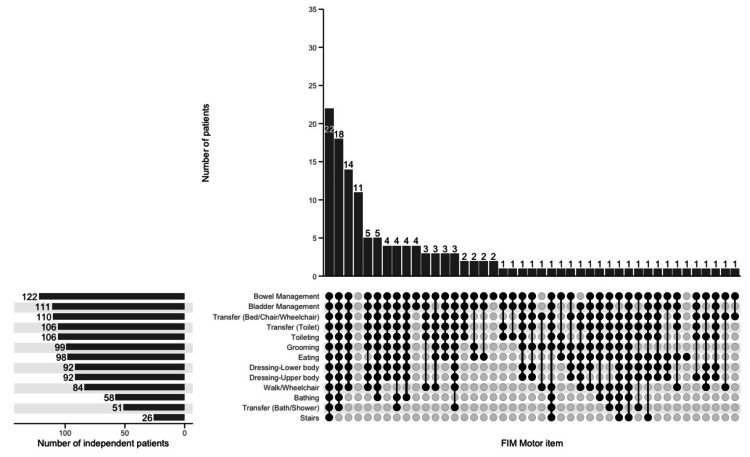
Combinations of motor FIM independence in home discharge (n = 136) The UpSet plot visualizes the distribution of independence combinations across motor FIM items. The bar chart at the top shows the frequency of each combination. The bar chart on the left displays the total number of independent patients for each FIM item. The dot matrix at the bottom indicates specific combinations of independent items, whereas connected black dots represent items that are simultaneously independent. FIM, Functional independence measure

Analysis of motor FIM item independence combinations using UpSet plots revealed four major combinations (defined as occurring in more than 10 cases or 7% of the 136 cases) in the home discharge group: independence in all 13 items (22 patients, or 16.2%), independence in 12 items excluding stairs (18 patients, or 13.2%), independence in 10 items excluding bathing, tub transfer, and stairs (14 patients, or 10.3%), and dependence in all items (11 patients, or 8.1%) (Figure 1). These four major combinations accounted for 47.8% of all cases.

**Figure 2:**
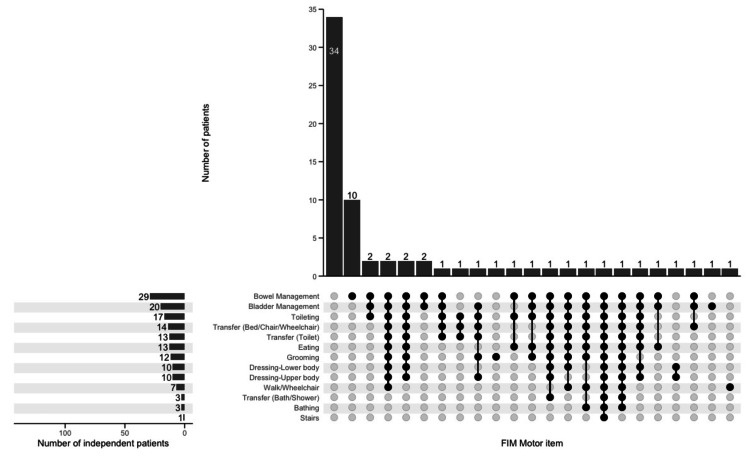
Combinations of motor FIM independence in non-home discharge (n = 69) Distribution of independence combinations across motor FIM items in the non-home discharge. FIM, Functional independence measure

In the non-home discharge group, the frequency of these same combinations was as follows: independence in all 13 items (one patient, or 1.4%), independence in 12 items excluding stairs (zero patients, or 0%), independence in 10 items excluding bathing, tub transfer, and stairs (two patients, or 2.9%), and dependence in all items (34 patients, or 49.3%) (Figure 2).

## Discussion

This study investigated the relationship between independence in motor FIM items at discharge and home discharge outcomes among stroke patients in convalescent rehabilitation wards. Using UpSet plots, we visualized combinations of independent motor FIM items and identified characteristic patterns associated with home discharge.

The UpSet plot analysis revealed key combinations of independent motor FIM items in the home discharge group. The main combinations included independence in all 13 items (22 patients, or 16.2%), independence in 12 items excluding stairs (18 patients, or 13.2%), and independence in 10 items excluding bathing, tub transfer, and stairs (14 patients, or 10.3%). We also identified cases with dependence in all items (11 patients, or 8.1%). These findings indicate that, while higher levels of ADL independence increase the likelihood of home discharge, complete independence is not a prerequisite.

The most frequent combination - independence in all items - demonstrates that comprehensive ADL independence is a significant facilitating factor for home discharge. This finding aligns with previous studies reporting motor FIM cutoff scores (56.5-60 points) necessary for home discharge [5,14,25]. In our study, complete independence in all items corresponds to a minimum score of 78 points (modified independence score of 6 points × 13 items), well above these previously reported cutoff values, supporting the feasibility of home discharge. We also identified a notable combination where patients were independent in all items except stairs, tub transfer, and bathing. These three items are known to be particularly challenging ADL tasks for stroke patients [21,26]. However, bathing assistance can be managed through long-term care insurance services [27], and stair-related issues can be addressed through home modifications [28]. Therefore, dependence on these three items does not preclude home discharge when patients are independent in other areas.

Our study found that some patients achieved home discharge despite being dependent in all items. This finding suggests that successful home discharge depends not only on patients' physical function but also on the caregiving capacity of family members and environmental modifications, which aligns with previous studies [4,6,12,29].

This study has several limitations. First, we did not include data on social background and detailed cognitive function in our analysis. Cognitive function is a crucial factor affecting both ADL performance and caregiver burden, so future studies should consider these factors along with housing environment and caregiving capacity. Second, we excluded severe cases and patients with complications. In particular, cases involving acute exacerbation transfers or severe dementia require a separate investigation. Third, this study was conducted at a single facility. As institutional characteristics and regional factors influence patient profiles and discharge destinations, caution is needed when generalizing these results.

Despite these limitations, our study successfully used UpSet plots to visually demonstrate combinations of independent motor FIM items. This approach revealed comprehensive patterns of independent items that were difficult to identify using conventional statistical methods. Our findings provide specific guidance for determining rehabilitation strategies and explaining outcomes to patients and their families in convalescent rehabilitation settings. The evidence that home discharge is possible even when patients are dependent on activities such as bathing and stair climbing provides scientific support for recommending long-term care services and environmental modifications. These results contribute to developing appropriate care service plans tailored to each patient's capabilities.

## Conclusions

This study analyzed the combinations of motor FIM independence associated with home discharge among stroke patients using UpSet plots. While complete independence in all motor FIM items was most common (16.2%) among discharged patients, successful home discharge was also achieved in cases with independence in 12 items, except for stairs (13.2%), and in cases with independence in 10 items, except for bathing, tub transfer, and stairs (10.3%), including cases with complete motor dependence (8.1%). The analysis revealed that dependence on specific activities, like bathing, tub transfer, and stair climbing, did not prevent home discharge when appropriate environmental modifications and care services were provided. These findings, visualized through UpSet plots - an approach that would be difficult to identify using conventional statistical methods - provide practical insights for developing rehabilitation strategies and care service plans based on patients' functional independence combinations.
